# Unlocking the Optimal Analgesic Potential: A Systematic Review and Meta-Analysis Comparing Intravenous, Oral, and Rectal Paracetamol in Equivalent Doses

**DOI:** 10.7759/cureus.41876

**Published:** 2023-07-14

**Authors:** Tarek Ibrahim, Amr Gebril, Mohammed K Nasr, Abdul Samad, Hany A Zaki

**Affiliations:** 1 Emergency, NMC Specialty Hospital, Al Ain, ARE; 2 Emergency Medicine, NMC Royal Hospital, Khalifa City, ARE; 3 Emergency Medicine, Dr. Sulaiman Al Habib Hospital, Dubai, ARE; 4 Acute Medicine/Emergency, NMC Royal Hospital, Khalifa City, ARE; 5 Emergency Medicine, Hamad Medical Corporation, Doha, QAT

**Keywords:** surgery, postoperative, pain scores, pain reduction, analgesia, acetaminophen, intravenous paracetamol, rectal, oral, parenteral

## Abstract

Paracetamol (acetaminophen) is an extensively used analgesic for acute and chronic pain management. Currently, paracetamol is manufactured for oral, rectal, and intravenous (IV) use. Research has shown varied results on the analgesic properties of IV paracetamol compared to oral and rectal paracetamol; however, research on the same doses of paracetamol is limited. Therefore, this review was constructed to explore the analgesic properties of IV paracetamol compared with oral and rectal paracetamol administered in equivalent doses.

A broad and thorough literature search was performed on five electronic databases, including PubMed, ScienceDirect, Medline, Scopus, and Google Scholar. Statistical analysis of all outcomes in our review was then performed using the Review Manager software. Outcomes were categorized as primary (pain relief and time to request rescue analgesia) and secondary (adverse events after analgesia). An extensive quality appraisal was also done using the Review Manager software’s Cochrane risk of bias tool.

The literature survey yielded 2,945 articles, of which 12 were used for review and analysis. The pooled analysis for patients undergoing surgical procedures showed that IV paracetamol had statistically similar postoperative pain scores at two (mean difference (MD) = -0.14; 95% confidence interval (CI) -0.58-0.29; p = 0.51), 24 (MD = 0.09; 95% CI = -0.02-0.21; p = 0.12), and 48 (MD = 0.04; 95% CI = -0.08-0.16; p = 0.52) hours as oral paracetamol. Similarly, the data on time to rescue analgesia showed no considerable difference between the IV and oral paracetamol groups (MD = -1.58; 95% CI = -5.51-2.35; p = 0.43). On the other hand, the pooled analysis for patients presenting non-surgical acute pain showed no significant difference in the mean pain scores between patients treated with IV and oral paracetamol (MD = -0.35; 95% CI = -2.19-1.48; p = 0.71). Furthermore, a subgroup analysis of analgesia-related adverse events showed that the incidences of vomiting/nausea and pruritus did not differ between patients receiving IV and oral paracetamol (odds ratio (OR) = 0.71; 95% CI = 0.45-1.11; p = 0.13 and OR = 0.48; 95% CI = 0.18-1.29; p = 0.05, respectively). A review of information from two trials comparing equal doses of IV and rectal paracetamol suggested that the postoperative pain scores were statistically similar between the groups.

IV paracetamol is not superior to oral or rectal paracetamol administered in equal doses. Therefore, we cannot recommend or refute IV paracetamol as the first-line analgesia for acute and postoperative pain.

## Introduction and background

Paracetamol, or acetaminophen, is an analgesic agent used extensively to manage acute and chronic pain. It is one of the most commonly used analgesic agents worldwide, with nearly 6,300 tonnes sold annually in the United Kingdom alone [1]. Research shows that paracetamol as an analgesic is safe and effective. For example, a previous Cochrane systematic review of oral paracetamol reported that out of 4,186 patients needing treatment, at least 50% achieved pain relief over four to six hours after administering a single paracetamol [2]. Side effects after paracetamol are rare, with therapeutic doses associated with extremely low incidences of liver failure (fewer than 1 in 500,000) [3]. Moreover, paracetamol only has two contraindications, one being paracetamol hypersensitivity and the other severe hepatocellular insufficiency.

Previously, paracetamol was manufactured for oral and rectal use; however, an intravenous (IV) formulation has also been available. A previous systematic review comparing the analgesic efficacy of IV paracetamol to oral paracetamol reported that IV paracetamol offered better postoperative pain than oral paracetamol [4]. On the contrary, Mallama and colleagues found no difference in postoperative pain between the IV and oral paracetamol groups [5]. On the other hand, a randomized trial comparing pain scores between IV and rectal acetaminophen in children undergoing tonsillectomy revealed that rectal administration was more effective in relieving pain than IV administration [6]. However, it is worth noting that all these studies used varying doses of paracetamol. Therefore, we designed this meta-analysis to determine whether IV paracetamol administered in equivalent dosages as oral or rectal paracetamol would have better analgesic properties. To answer this question, we evaluated the pain scores after surgical operations and in patients presenting with non-surgical acute pain.

## Review

Methodology

Literature Search

Two reviewers independently conducted a broad literature survey on five electronic databases (i.e., PubMed, Scopus, ScienceDirect, Medline, and Google Scholar). In the search, the reviewers identified all articles published until May 2023 using the following keywords and MeSH terms: (Intravenous OR IV OR parenteral) AND (Oral) AND (Rectal) AND (paracetamol OR acetaminophen) AND (analgesia OR pain reduction OR pain scores OR pain relief) AND (postoperative OR surgery) AND (non-surgical acute pain).

Eligibility Criteria

One independent reviewer was tasked with deriving the criteria for including and excluding articles for this study. After an extensive analysis of the topic, the reviewer framed the following inclusion criteria: randomized trials and observational studies published in the English language; studies that directly compared outcomes for IV paracetamol with either oral or rectal paracetamol; studies having at least one of the following outcomes: pain scores, time to rescue analgesia, or incidences of adverse events after analgesia; studies where paracetamol was administered either preoperatively, postoperatively, or intraoperatively; and studies where paracetamol was used for non-surgical acute pain. Moreover, during surgical operations, opioids in combination with other anesthetic agents are used to induce anesthesia. Similarly, intravenous opioids are usually considered first-line analgesics for patients presenting with non-surgical severe pain; therefore, we included articles where paracetamol was administered after opioid administration.

On the other hand, the reviewer framed the following criteria to exclude articles from review and analysis: studies that compared antipyretic effects of IV paracetamol to oral or rectal paracetamol, studies in which IV paracetamol was not offered in the same dosage as either oral or rectal paracetamol, and all close or exact duplicates and unpublished data.

Data Extraction and Definitions

Two independent reviewers screened all included articles and retrieved the data needed for review and analysis. The data collected by the reviewers included author ID (the first author’s last name and year of publication), study design, location (country) of the study, characteristics of the participants (sample size, gender distribution, and mean/median age), the dosage used for analgesia, cause of pain, and the primary outcomes. Outcomes in our meta-analysis were then categorized into primary and secondary. The preliminary results were postoperative or acute pain relief and time to rescue analgesia, while the secondary outcome was incidences of adverse events after analgesia. In the event of discrepancies in the collected data, the two reviewers held a constructive discussion. If they could not reach a consensus, a third reviewer was consulted.

Data Analysis

All meta-analyses in this study were performed using the Review Manager software (RevMan 5.4.1). The DerSimonian & Laird, random-effects model was employed in the analyses to counter the anticipated heterogeneity and provide conservative pooled effect sizes. Moreover, outcomes related to pain and time to rescue were continuous; therefore, the overall effect sizes were calculated using mean differences (MD). On the other hand, adverse effect outcomes were dichotomous; therefore, the effect sizes were calculated using the simple odds ratio (OR). If the interquartile ranges (IQR), 95% confidence intervals (CIs), and range were provided instead of the standard deviation (SD), then online calculators were used. For conversion of range to SD, the difference between the range was divided by 4 for sample sizes between 16 and 70 and by 6 for larger sample sizes [7], while for conversion of IQR to SD, the difference was divided by 1.35 [8]. The 95% CIs were converted to SD using the formula SD = √N ⅹ (upper limit - lower limit)/3.92 [9]. In addition, pain scores measured using the 100 mm scale were converted to the 10 cm pain scale. Heterogeneity between studies was also calculated using the I^2^ statistics, and considered values of 0-49, 50-69, and 70-100 as low, moderate, and high, respectively. During the analyses, a 95% CI was chosen, and statistical significance was defined by p-values <0.05.

Quality Appraisal

**Figure 1:**
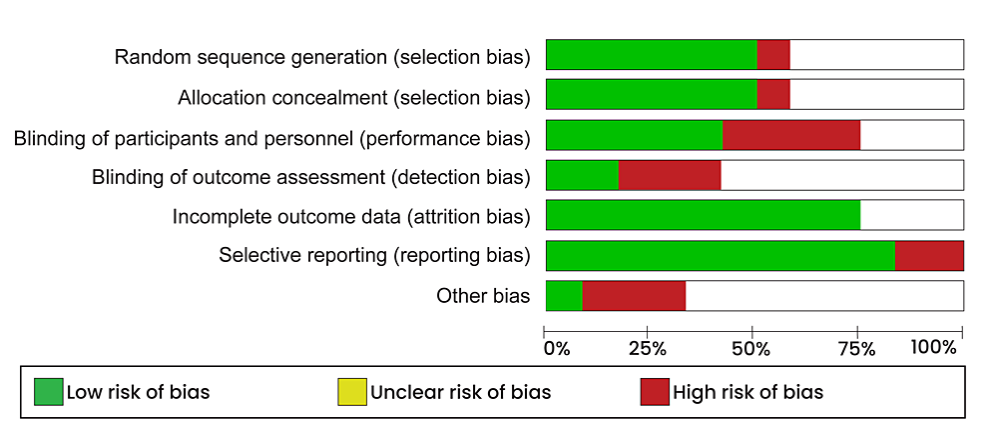
Risk of bias graph.

**Figure 2:**
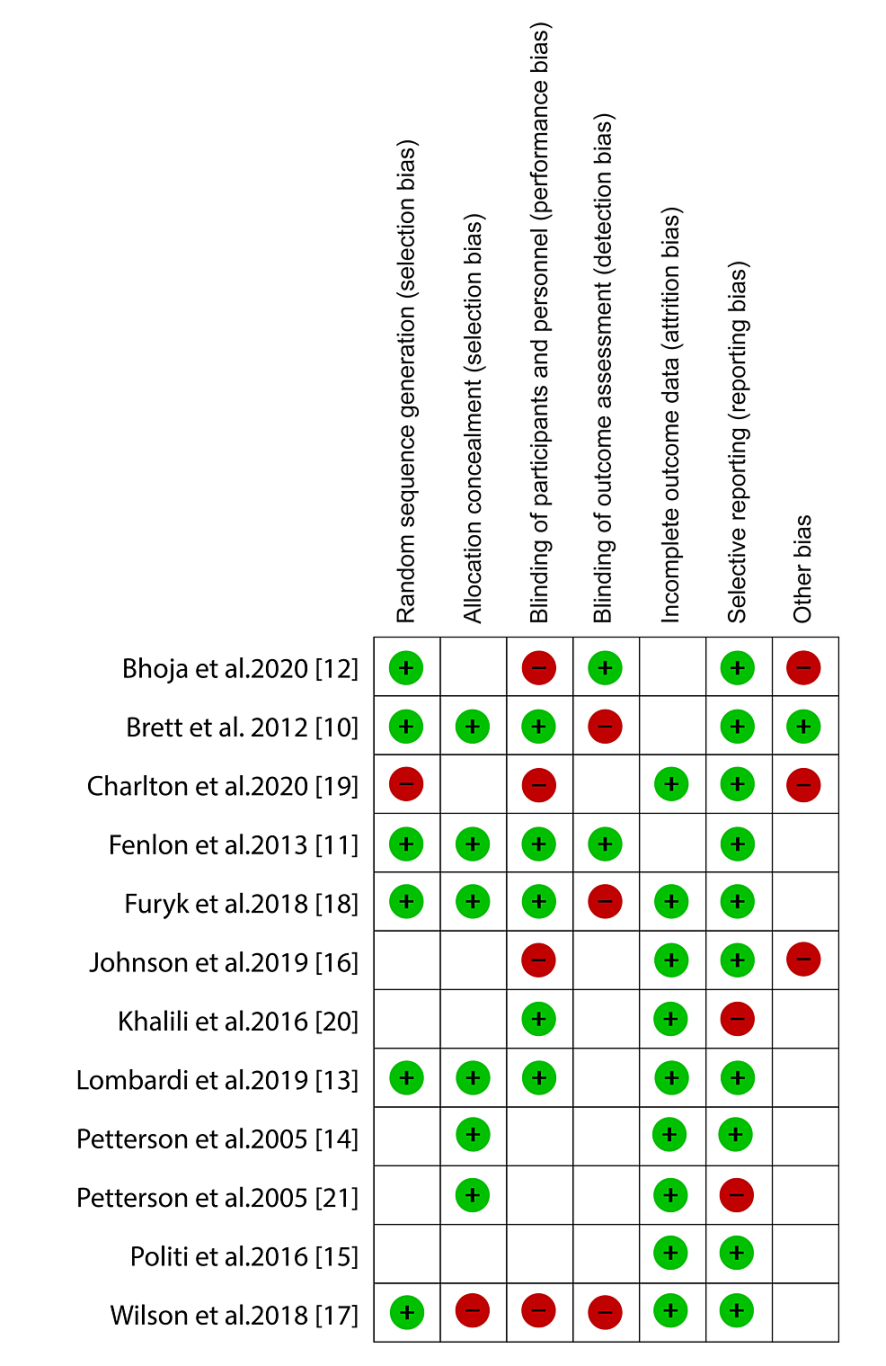
Risk of bias summary. Brett et al. (2012) [10], Fenlon et al. (2013) [11], Bhoja et al. (2020) [12], Lombardi et al. (2019) [13], Petterson et al. (2005) [14], Politi et al. (2016) [15], Johnson et al. (2019) [16], Wilson et al. (2018) [17], Furyk et al. (2018) [18], Charlton et al. (2020) [19], Khalili et al. (2016) [20], Petterson et al. (2005) [21].

One experienced reviewer was tasked with the quality appraisal process. The reviewer used Cochrane’s risk of bias tool in the Review Manager software to perform the quality appraisal. Using this tool, he assessed each study’s selection, performance, attrition, and reporting bias. Afterward, the quality of studies was determined by converting the risk of bias scores to the Agency of Healthcare Research Quality Standards. The risk of bias assessment results in the form of the risk of bias graph is shown in Figure 1. A risk of bias summary is presented in Figure 2.

Results

Study Selection

**Figure 3:**
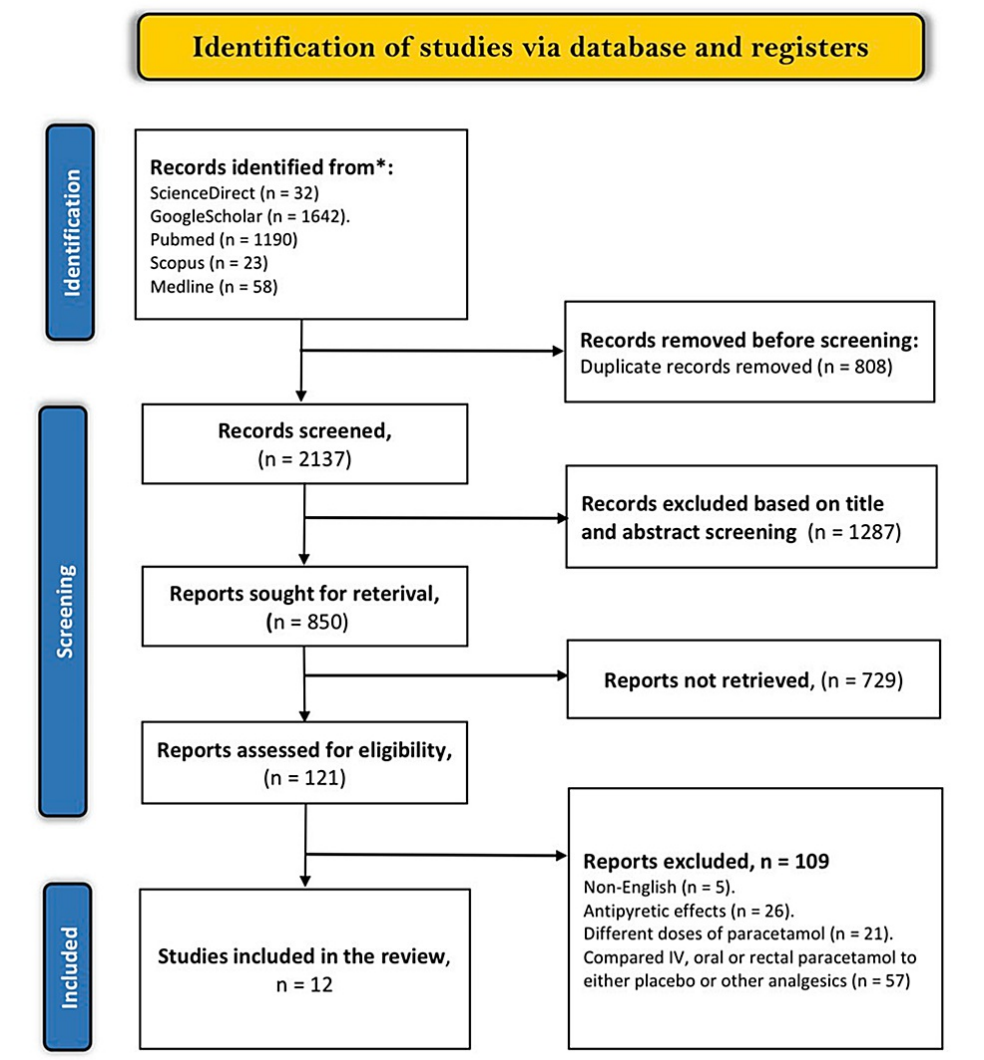
PRISMA flow diagram of the literature search results. PRISMA = Preferred Reporting Items for Systematic Reviews and Meta-Analyses

Using the MeSH terms and keywords mentioned earlier, the reviewers accumulated 2,945 articles from the electronic databases. A duplicate evaluation of these articles excluded 808 articles deemed close or exact duplicates. Afterward, the reviewers screened the titles and abstracts of the remaining articles and found that 1,287 did not meet the screening criteria. Of the remaining 850 articles, the reviewers did not retrieve 729 because they were either ongoing trials, conference abstracts, systematic reviews, letters to the editor, case reports, or case series. Finally, only 12 articles were eligible for inclusion, while the other 109 were excluded for the following reasons: five were published in different languages; 57 compared either IV, oral, or rectal paracetamol to placebo or other analgesics; 26 compared antipyretic effects of IV paracetamol to oral or rectal paracetamol; and 21 compared different doses of IV, oral, and rectal paracetamol. The Preferred Reporting Items for Systematic Reviews and Meta-Analyses flow diagram presents the full selection criteria (Figure 3). A summary of study characteristics is shown in Table 1.

**Table 1:** Summary of study characteristics. RCT = randomized controlled trial; IV = intravenous

Author ID	Study design	Location	Patients’ characteristics	Dose	Source of pain	Main outcomes	Overall quality
Brett et al. (2012) [10]	A double-blind RCT	New Zealand	30 patients (11 females and 19 males)	1.0 g oral paracetamol 3–60 minutes preoperatively and 1.0 g paracetamol intraoperatively	Knee arthroscopy	Patients receiving oral paracetamol required more rescue opioid analgesia than those receiving IV paracetamol; however, the difference was insignificant (45.5 (85.0) vs. 14 (19.6) µg; p = 0.53). Mean pain scores were similar for patients receiving IV and oral paracetamol (15.3 (3.1) vs. 23.1 (4.7); p = 0.17)	Fair
Fenlon et al. (2013) [11]	A double-blind RCT	The United Kingdom	128 patients (34 males and 94 females; ages: 18–65 years)	1.0 g oral and IV paracetamol preoperatively	Lower third molar extractions	The mean pain scores did not differ between the oral and IV groups (5.2 (2.2) vs. 4.7 (2.2), respectively). Nine of 63 patients receiving IV paracetamol required rescue analgesia, while 18 in the oral group required rescue analgesia	Fair
Bhoja et al. (2020) [12]	A single-center prospective RCT	The United States	101 patients (33 females and 68 males; mean age: 52.7 (16.0) years)	1,000 mg oral paracetamol preoperatively and 1,000 mg IV paracetamol intraoperatively	Sinus surgery	The main pain scores 1 hour after the operation were statistically similar between the IV and oral groups (2.83 (2.55) vs. 2.13 (1.93); p = 0.252). No difference was recorded in the need for opioid rescue analgesia between patients receiving IV and oral opioids (5.0 (0.0, 14.8) vs. 5.0 (0.0, 10.0), respectively). The incidences of nausea and vomiting post-operation did not differ between the IV and oral groups (p = 0.620 and p = 0.495, respectively)	Poor
Lombardi et al. (2019) [13]	A double-blind RCT	The United States	75 women	1 g oral and IV paracetamol preoperatively	Robotic-assisted laparoscopic hysterectomy	The mean pain scores 2 hours after the surgical operations did not differ between the IV and oral paracetamol groups (35 ± 2.7 vs. 36 ± 1.9; p = 0.86). Incidences of nausea were statistically similar after IV and oral paracetamol administration (7 (19%) vs. 14 (38%); p = 0.12). The use of rescue of opioid analgesics was statistically similar between the oral and IV paracetamol groups (9.7 ± 7.2 vs. 9.5 ± 7.2; p = 0.90)	Fair
Petterson et al. (2005) [14]	A prospective RCT	Sweden	77 patients (64 males and 13 females)	1 g oral and IV paracetamol every 6 hours postoperatively	Coronary artery bypass grafting	The incidence of postoperative nausea and vomiting was statistically similar between the oral and IV groups (19/38 vs. 13/39, respectively). The difference in mean pain scores between the IV and oral groups was statistically insignificant (2 (0–6) vs. 2 (0–5, respectively)	Fair
Politi et al. (2016) [15]	A prospective RCT	The United States	120 patients	1 g oral and IV paracetamol preoperatively and every 6 hours postoperatively	Hip and knee replacement surgery	The 24-hour mean pain scores between the IV and oral groups were statistically similar (3.0 vs. 3.40; p = 0.06). The consumption of opioids 24 hours after the operation did not differ between the IV and oral groups (3.48 vs. 3.71; p = 0.76)	Fair
Johnson et al. (2019) [16]	Retrospective analysis	The United States	579 patients (44 males and 535 females)	1,000 mg oral paracetamol preoperatively and 1,000 mg paracetamol intraoperatively	Elective laparoscopic cholecystectomies	The overall mean pain scores were statistically similar for patients receiving oral and IV paracetamol (2 (0, 3) vs. 2 (0, 3), respectively). The time to rescue analgesia was shorter in the IV group as opposed to the oral group (21 vs. 23 minutes; p = 0.014)	Poor
Wilson et al. (2018) [17]	Single-centre, prospective, three-arm, non-blinded RCT	The United States	141 female patients	1 g IV and oral paracetamol every 8 hours postoperatively	Cesarean delivery	The average pain scores were not different for patients receiving oral and IV paracetamol (23.8 (3.00) vs. 24.2 (2.98), respectively). The time until the first opioid rescue analgesia did not differ between the oral and IV groups (24.0 (26.3) vs. 25.3 (8.5), respectively). Incidences of nausea, vomiting, and pruritus were similar for oral and IV groups (31.9% vs. 34.0%, 8.51% vs. 4.26%, and 29.8% vs.17.0%, respectively)	Poor
Furyk et al. (2018) [18]	A single-center, double-blinded RCT	Australia	87 patients (52 females and 35 males)	1 g IV and oral paracetamol	Acute pain	The average pain scores were similar throughout the study (p = 0.86, 0.62, 0.52, 060, 0.28, 0.72, and 0.56 at 15, 30, 45, 60, 120, 180, and 240-minute intervals, respectively). The need for rescue opioid analgesia was similar between the IV and oral groups (86.2% vs. 84.1%, p =0.69). The average opioid consumption was statistically similar (5mg (2.5–5) vs. 5mg (2.5–5.0) for the oral and IV groups, respectively)	Fair
Charlton et al. (2020) [19]	Case-control study	The United Kingdom	80 patients (54 females and 26 males; mean age: 54 years ± 3 years)	1 g IV and oral paracetamol	Acute pain	IV paracetamol had a clinically significant improvement in pain scores than oral paracetamol (p = 0.013)	Poor
Khalili et al. (2016) [20]	Prospective, double-blind RCT	Iran	120 patients (104 males and 16 females; Ages: 6 months to 6 years)	15 mg/kg IV and rectal paracetamol	Inguinal herniorrhaphy	The mean pain scores significantly differed between IV and rectal groups at 30 minutes and 1 and 2 hours after extubating (p < 0.0001)	Fair
Petterson et al. (2005) [21]	A prospective RCT	Sweden	35 patients (20 women and 15 men; median age: 49 (20–72) years)	1 g IV paracetamol and 1–2 g oral or rectal paracetamol	Ambulatory surgery	The need for rescue analgesia was recorded in one patient receiving IV paracetamol and none in the 1 g oral or rectal groups. Nausea was recorded in three patients in the IV group and one patient in each of the 1 g oral and rectal groups	Fair

In Patients Undergoing Surgical Procedures, Does Intravenous Paracetamol Have Better Analgesic Properties Than Oral Paracetamol in Equivalent Doses?

**Figure 4:**
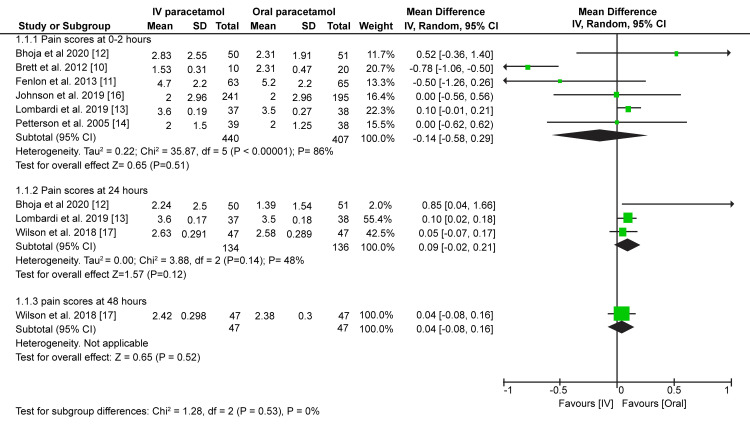
Forest plot prevalence and 95% confidence interval comparing postoperative pain scores between intravenous and oral paracetamol groups. Brett et al. (2012) [10], Fenlon et al. (2013) [11], Bhoja et al. (2020) [12], Lombardi et al. (2019) [13], Petterson et al. (2005) [14], Johnson et al. (2019) [16], Wilson et al. (2018) [17].

Despite paracetamol being a widely used analgesic, its benefit in an IV form over an oral form remains unclear. Therefore, in this study, we pooled data to compare the analgesic properties of IV paracetamol to oral paracetamol administered in the same doses. The analgesic properties were assessed by comparing postoperative pain scores and the time to rescue analgesia was administered. Data pooled from six studies showed no considerable difference in the postoperative pain scores after two hours in the recovery units between the IV and oral paracetamol groups (MD = -0.14; 95% CI = -0.58-0.29; p = 0.51) (Figure 4). Moreover, our meta-analysis did not find any considerable difference in postoperative pain scores recorded at 24 hours (MD = 0.09; 95% CI = -0.02-0.21; p = 0.12) and 48 hours (MD = 0.04; 95% CI = -0.08-0.16; p = 0.52) (Figure 4).

**Figure 5:**
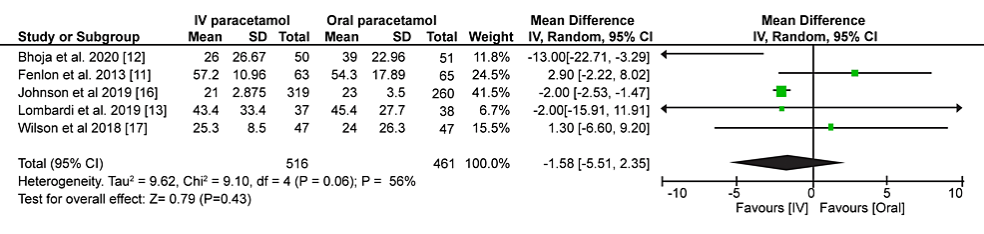
Forest plot prevalence and 95% confidence interval comparing time to rescue analgesia between intravenous and oral paracetamol groups. Fenlon et al. (2013) [11], Bhoja et al. (2020) [12], Lombardi et al. (2019) [13], Johnson et al. (2019) [16], Wilson et al. (2018) [17].

On the other hand, data from five trials suggested that the time taken to request the first rescue analgesia is shorter in patients receiving IV paracetamol than oral paracetamol. However, the difference between the two groups was not clinically significant (MD = -1.58; 95% CI = -5.51-2.35; p = 0.43) (Figure 5).

In Patients With Non-surgical Acute Pain, Does Intravenous Paracetamol Have Better Analgesic Properties Than Oral Paracetamol in Equivalent Doses?

**Figure 6:**
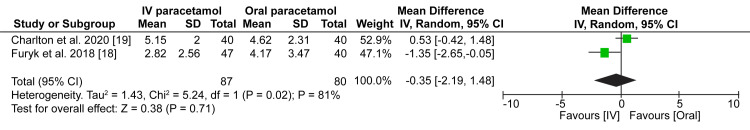
Forest plot prevalence and 95% confidence interval comparing the mean pain scores for patients with acute pain receiving intravenous and oral paracetamol. Furyk et al. (2018) [18], Charlton et al. (2020) [19].

Most of the research has been centered around the postoperative analgesic effects of paracetamol; however, evidence shows that paracetamol is also used for analgesia in patients with non-surgical acute pain. Data pooled from two articles comparing the analgesic efficacy of paracetamol in patients with acute pain showed no considerable difference in the average pain scores between patients receiving IV and oral paracetamol (MD = -0.35; 95% CI = -2.19-1.48; p = 0.71) (Figure 6).

Is Intravenous Paracetamol Safe as Oral Paracetamol in Equivalent Doses?

**Figure 7:**
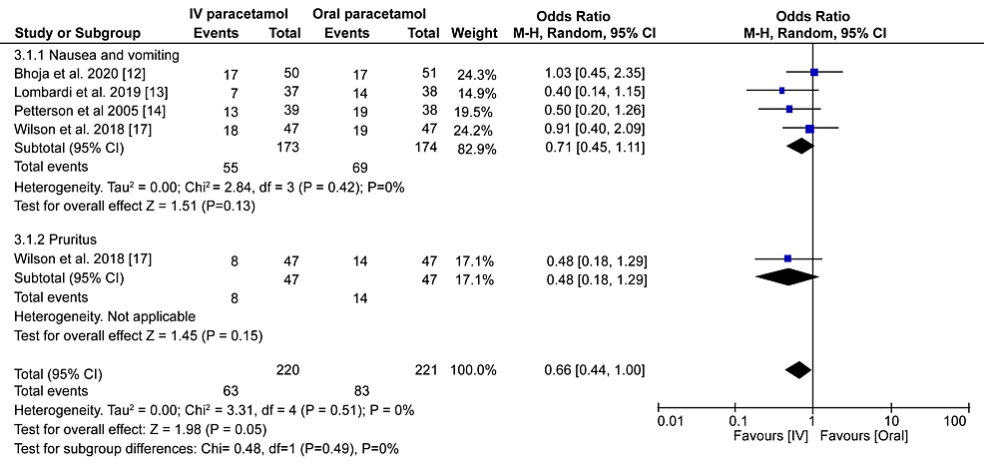
Forest plot prevalence and 95% confidence interval comparing adverse events between intravenous and oral paracetamol groups. Bhoja et al. (2020) [12], Lombardi et al. (2019) [13], Petterson et al. (2005) [14], Wilson et al. (2018) [17].

To analyze whether IV paracetamol is as safe as oral paracetamol, we studied the incidences of vomiting/nausea and pruritus. Our meta-analysis of four randomized trials showed that the incidence of nausea/vomiting and pruritus did not differ between the IV and oral paracetamol groups (OR = 0.71; 95% CI = 0.45-1.11; p = 0.13 and OR: 0.48; 95% CI = 0.18-1.29; p = 0.05, respectively) (Figure 7).

Does Intravenous Paracetamol Have Better Analgesic Effects Than Rectal Paracetamol in Equivalent Doses?

Only two studies in this article compared the analgesic efficacy of IV paracetamol to rectal paracetamol administered in the same dosage. However, due to poorly reported outcomes, meta-analyses could not be performed. Khalili et al. [20] compared the analgesic efficacy of 15 mg/kg of IV paracetamol to 15 mg/kg of rectal paracetamol in pediatric patients undergoing inguinal herniorrhaphy and found that the pain scores were low for patients receiving IV or rectal paracetamol. However, the investigators of the trial did not record any considerable difference between the groups over the 12-hour study period (p > 0.05). On the other hand, Pettersson and colleagues compared the bioavailability of oral, rectal, and IV paracetamol after surgery [21]. They found that the pain scores between 1 g IV, oral, and rectal paracetamol did not differ statistically (p > 0.05).

Discussion

There is limited research on the analgesic properties of equal doses of IV, oral, and rectal paracetamol. Therefore, this is the first systematic review to explore the analgesic effects of IV, oral, and rectal paracetamol offered in equivalent doses and form a basis for emergency care. Our analysis has shown that IV paracetamol has a similar effect on postoperative and non-surgical acute pain reduction as oral paracetamol in equal doses. Similarly, we found no considerable difference in the time until the first request for opioid rescue analgesia. Moreover, IV paracetamol seems to have similar analgesic effects as rectal paracetamol offered in equivalent doses.

Our findings on postoperative pain scores at two, 24, and 48 hours after surgical procedures are collaborated by a previous systematic review of two randomized controlled trials (RCTs) comparing the same dose of IV and oral paracetamol in patients undergoing knee and hip arthroplasty [22]. According to the analysis from the review, patients receiving IV paracetamol had similar pain scores as those receiving oral paracetamol at 12, 24, and 48 hours after arthroplasties (p = 0.716, 0.495, and 0.621, respectively). This insignificant difference in postoperative pain scores in our study can be explained by the fact that all studies on surgical pain included patients who had received opioids during induction of analgesia, implying that these patients already had achieved some degree of pain relief at baseline. Contrary to our findings, evidence suggests that IV paracetamol may offer pain relief faster than oral paracetamol. Politi and colleagues reported that during the first four-hour interval (0-4 hours), postoperative pain scores were lower in the IV group compared to the oral group (p = 0.03) [15]. However, the difference was only one point which might not be considered clinically significant. Moreover, the study had some methodological limitations that might have influenced the results. First, the study had a small sample size, meaning potential significant differences could not be achieved throughout the 24-hour study period. Furthermore, the study did not state whether the patients or healthcare personnel were blinded to the treatment, meaning there could have been a bias toward the IV group. Finally, this trial was conducted in a single center and may not be a true representation of the entire population.

Our meta-analysis also demonstrated that IV paracetamol offered in the same dose as oral paracetamol does not significantly benefit patients with non-surgical acute pain. However, one study included in this analysis reported that IV paracetamol is more advantageous than oral paracetamol in managing pain in out-of-hospital settings [19]. This significant difference can be attributed to how the body absorbs paracetamol intravenously. In their research, Brett and colleagues evaluated postoperative plasma levels and found that all patients assigned to the IV paracetamol group achieved plasma levels above the analgesic levels, while less than half the patients receiving oral paracetamol had achieved the same plasma levels [10]. Given that plasma concentrations are well known to reflect the therapeutic and analgesic effects of analgesics, it explains the significant difference in that study. Furthermore, the study included patients who did not receive opioids before paracetamol administration, meaning that there was no pain relief at baseline. However, the findings of this article cannot be used primarily to guide the clinical use of IV paracetamol in all patients presenting with acute pain in an out-of-hospital setting due to various limitations. First, this study was statistically underpowered as it had a low sample size. Moreover, the paramedics were not blinded to the treatment, and the investigators could not account for the preference for IV paracetamol among the paramedics, meaning that observer bias was introduced in their results. Besides, the study used the Numerical Rating Scale (NRS) to evaluate pain scores. This pain scale is usually limited as it cannot accommodate patients who cannot score their pain verbally, and it does not always capture the full complexity of pain. Additionally, the study had an observational design; thus, it was subject to bias and confounding.

When comparing analgesic efficacy, the timing of rescue analgesia is also an important measure. Our research shows that time to rescue analgesia does not differ between patients receiving IV and oral paracetamol. However, evidence suggests that IV paracetamol patients might request rescue analgesia earlier than those receiving oral paracetamol [16]. In their research, Johnson and colleagues found that IV paracetamol among patients undergoing laparoscopic cholecystectomy was associated with a shorter time to request rescue analgesia than oral paracetamol (p = 0.014) [16]. However, the difference between the two groups was only two minutes which might be clinically questionable and hinders the suggestion that oral paracetamol may provide better analgesic effects than IV paracetamol. Moreover, the results of this study present some methodological concerns. The first concern is that the study was designed as a retrospective analysis with low methodological quality. Second, the healthcare providers were not blinded to the treatment protocols, thus introducing observer bias to their results. In addition, it lacked strict treatment protocols, which might have seen patients crossing over to other treatment arms.

The rescue opioid analgesia required is also substantial when comparing the analgesic efficacy of IV and oral paracetamol. In our study, we could not pool the data for the amount of rescue analgesia as different measurements were used in each study. Furyk et al. [18] measured the amount of rescue opioid analgesia in morphine equivalents and found no difference between the IV and oral groups (5 mg (2.5-5) vs. 5 mg (2.5-5.0); p = 0.50). Similarly, Lombardi and colleagues found no clinical difference in the total opioid consumption as measured in morphine equivalents between the oral and IV groups (p = 0.90) [13]. On the other hand, Politi and colleagues measured the rescue opioid consumption in hydromorphone equivalents. They found no considerable difference between the IV and oral groups over the 24-hour study period (p = 0.76) [15]. Following the results presented in these research articles, the consumption of rescue opioid analgesia does not improve or decline with IV paracetamol utilization as opposed to oral paracetamol.

Although we have shown that IV paracetamol offers similar analgesic effects as oral paracetamol in equivalent doses, the preferred use of paracetamol in IV form over oral form is due to various reasons. Foremost, gastric emptying and therapy uptake of oral drugs is usually slowed by the concomitant use of opioids [23,24]. Next, preoperative fasting and long-lasting analgesia are known to affect the uptake of drugs administered in oral form. Therefore, oral administration of analgesia results in a wide range of individual plasma concentrations [25]. Likewise, research has shown that paracetamol in an IV form has faster-onset analgesia than when administered orally. For example, Moller and colleagues reported that IV paracetamol after the third molar surgery had reduced the time taken to achieve meaningful pain relief (8 minutes vs. 37 minutes for IV and oral paracetamol, respectively) and maximal pain relief (15 minutes vs. 1 hour, for IV and oral paracetamol, respectively) [26].

However, this preferred use of IV paracetamol may be precluded due to several factors. IV paracetamol may present risks such as local infections and phlebitis. Therefore, similar to oral paracetamol, IV paracetamol should be cautiously provided to patients with known risks of hepatoxicity, and the doses should be adjusted for patients with low body weight [27]. IV paracetamol is also associated with increased costs compared to oral and rectal paracetamol. This is evident in an Australian report that 1,000 mg of IV paracetamol costs around $3.30 while the cost of two 500 mg oral tablets or rectal suppositories cost $0.026 and $1.24, respectively [28]. Similarly, Bhoja and colleagues estimated a single dose of IV paracetamol costs $30, while a single dose of oral paracetamol costs about $0.01 [12].

The data review from two articles also suggested that IV paracetamol has similar analgesic effects as rectal paracetamol in equivalent doses. However, research shows that higher doses of rectal paracetamol have better analgesic properties than IV paracetamol. Mahajan et al. [29] compared 35-45 mg/kg of rectal paracetamol to IV paracetamol infused at a rate of 10-15 mg/kg. They found that despite the groups’ postoperative pain scores being comparable, those receiving rectal paracetamol needed less rescue analgesia and spent longer on analgesia before requiring rescue analgesia. In contrast, other research has shown no superiority of IV paracetamol over rectal paracetamol, irrespective of the dose. For example, Soyer and colleagues compared 40 mg/kg of rectal paracetamol to 15 mg/kg of IV paracetamol among pediatric patients undergoing circumcision. They found no considerable difference in postoperative pain (p > 0.05) [30]. Moreover, the trial demonstrated an insignificant difference in the number of patients requiring rescue analgesia (24 (63.15%) vs. 18 (25%) for rectal and IV paracetamol groups, respectively). The evidence from these studies implies that higher doses of rectal paracetamol have varied analgesic effects compared to IV paracetamol; therefore, future research should focus on the analgesic efficacy of higher doses of rectal paracetamol.

While our research was focused on comparing IV paracetamol to oral or rectal paracetamol, evidence suggests that rectal paracetamol is less effective than oral paracetamol when administered at the same dose. Anderson et al. [31] studied the analgesic efficacy of paracetamol among children undergoing tonsillectomy. They found that patients receiving 40 mg/kg of oral paracetamol had lower median pain scores than those receiving 40 mg/kg of rectal paracetamol (p = 0.018). Moreover, the need for rescue morphine in the recovery room was significantly higher in the rectal group than in the oral group (23/50 vs. 10/50; p < 0.001). The outcomes of this should be interpreted with caution as it was statistically underpowered due to a small sample size. Therefore, more randomized trials with larger sample sizes are required to fully establish the outcomes reported in that trial.

Our study also found no considerable difference in the incidence of nausea/vomiting and pruritus between the IV and oral groups. However, these adverse effects are usually related to opioid consumption rather than the paracetamol administration. This is evident from our previous meta-analyses, where incidences of nausea and vomiting have been reported after using opioids for procedural and acute pain management [32,33]. Although none of the studies reported hypotension incidences, evidence suggests that IV paracetamol can induce hypotension. Boyle et al. [34] indicated that IV paracetamol administration in febrile patients is associated with reduced skin blood flow and hypotension. The authors concluded that the antipyretic effect of IV paracetamol causes the mechanism of hypotension.

Study limitations

Similar to any other systematic review, our research also had several limitations to consider when interpreting our results. Primarily, our eligibility criteria only allowed studies published in the English language to be used for review and analysis, meaning that all the relevant data and information that would have been used to improve our scientific and statistical analyses were disregarded. Moreover, we included articles where patients had received opioids before paracetamol administration, meaning that these patients had acquired some degree of pain relief at baseline, which might have affected our pain control results. Next, some of our meta-analysis results had substantial heterogeneity. However, this heterogeneity justified the use of the random-effect model. Moreover, heterogeneity was expected as the data used for postoperative pain management included varying surgical procedures and sample sizes. Third, we used observational studies and randomized trials that did not blind the patients or healthcare personnel in the analyses; therefore, the potential bias in these studies was transferred to our meta-analyses. Additionally, the conclusions on IV versus rectal paracetamol were made based on the information from two articles with poor outcome reporting; therefore, more detailed randomized trials are needed to establish our conclusion fully. Besides, some of the studies used the NRS to assess pain. Although this pain measuring scale is simple to use and well understood by most healthcare professionals, it fails to capture the full extent of pain, meaning that some pain outcomes might have been undermined and influenced by our statistical analyses. Finally, most studies used in the analyses were statically underpowered due to low sample sizes; therefore, our meta-analyses were also undermined.

## Conclusions

In summary, our study findings did not demonstrate any analgesic superiority of IV paracetamol compared to oral and rectal paracetamol administered in equivalent doses. Therefore, we cannot recommend or refute the use of IV paracetamol over oral or rectal paracetamol for acute and postoperative pain management. However, IV paracetamol might be recommended over oral paracetamol in patients with delayed gastric emptying or those unable to tolerate oral drugs.

## References

[1] Moore RA, Moore N (2016). Paracetamol and pain: the kiloton problem. Eur J Hosp Pharm.

[2] Barden J, Edwards J, Moore A, McQuay H (2004). Single dose oral paracetamol (acetaminophen) for postoperative pain. Cochrane Database Syst Rev.

[3] Sinatra RS, Jahr JS, Reynolds LW, Viscusi ER, Groudine SB, Payen-Champenois C (2005). Efficacy and safety of single and repeated administration of 1 gram intravenous acetaminophen injection (paracetamol) for pain management after major orthopedic surgery. Anesthesiology.

[4] Stagg K (2021). Intravenous versus oral paracetamol for postoperative analgesia: a systematic review. J Perioper Pract.

[5] Mallama M, Valencia A, Rijs K, Rietdijk WJ, Klimek M, Calvache JA (2021). A systematic review and trial sequential analysis of intravenous vs. oral peri-operative paracetamol. Anaesthesia.

[6] Kumar U, Kainat Kainat, Kumar R, Mahender Mahender, Khan B, Hiranand Hiranand (2022). Comparison of pain scores between intravenous versus rectal acetaminophen in children undergoing tonsillectomy. Pak J Med Health Sci.

[7] Hozo SP, Djulbegovic B, Hozo I (2005). Estimating the mean and variance from the median, range, and the size of a sample. BMC Med Res Methodol.

[8] (2023). 7.7.3.5 Medians and interquartile ranges. http://1.cochrane.org/chapter_7/7_7_3_5_mediansand_interquartile_ranges.htm.

[9] (2023). 7.7.3.2 Obtaining standard deviations from standard errors. http://1.cochrane.org/chapter_7/7_7_3_2_obtaining_standard_deviations_from_standard_errors_and.htm.

[10] Brett CN, Barnett SG, Pearson J (2012). Postoperative plasma paracetamol levels following oral or intravenous paracetamol administration: a double-blind randomised controlled trial. Anaesth Intensive Care.

[11] Fenlon S, Collyer J, Giles J (2013). Oral vs intravenous paracetamol for lower third molar extractions under general anaesthesia: is oral administration inferior?. Br J Anaesth.

[12] Bhoja R, Ryan MW, Klein K (2020). Intravenous vs oral acetaminophen in sinus surgery: a randomized clinical trial. Laryngoscope Investig Otolaryngol.

[13] Lombardi TM, Kahn BS, Tsai LJ, Waalen JM, Wachi N (2019). Preemptive oral compared with intravenous acetaminophen for postoperative pain after robotic-assisted laparoscopic hysterectomy: a randomized controlled trial. Obstet Gynecol.

[14] Pettersson PH, Jakobsson J, Owall A (2005). Intravenous acetaminophen reduced the use of opioids compared with oral administration after coronary artery bypass grafting. J Cardiothorac Vasc Anesth.

[15] Politi JR, Davis RL 2nd, Matrka AK (2017). Randomized prospective trial comparing the use of intravenous versus oral acetaminophen in total joint arthroplasty. J Arthroplasty.

[16] Johnson RJ, Nguyen DK, Acosta JM, O’Brien AL, Doyle PD, Medina-Rivera G (2019). Intravenous versus oral acetaminophen in ambulatory surgical center laparoscopic cholecystectomies: a retrospective analysis. P T.

[17] Wilson SH, Wolf BJ, Robinson SM, Nelson C, Hebbar L (2019). Intravenous vs oral acetaminophen for analgesia after cesarean delivery: a randomized trial. Pain Med.

[18] Furyk J, Levas D, Close B (2018). Intravenous versus oral paracetamol for acute pain in adults in the emergency department setting: a prospective, double-blind, double-dummy, randomised controlled trial. Emerg Med J.

[19] Charlton K, Limmer M, Moore H (2020). Intravenous versus oral paracetamol in a UK ambulance service: a case control study. Br Paramed J.

[20] Khalili GR, Shafa A, Yousefi R (2016). Comparison of the effects of preemptive intravenous and rectal acetaminophen on pain management after inguinal herniorrhaphy in children: a placebo-controlled study. Middle East J Anaesthesiol.

[21] Holmér Pettersson P, Hein A, Öwall A, Anderson RE, Jakobsson JG (2005). Early bioavailability in day surgery: a comparison between orally, rectally, and intravenously administered paracetamol. Ambulatory Surg.

[22] Sun L, Zhu X, Zou J, Li Y, Han W (2018). Comparison of intravenous and oral acetaminophen for pain control after total knee and hip arthroplasty: a systematic review and meta-analysis. Medicine (Baltimore).

[23] Mushambi MC, Rowbotham DJ, Bailey SM (1992). Gastric emptying after minor gynaecological surgery. The effect of anaesthetic technique. Anaesthesia.

[24] Kennedy JM, Tyers NM, Davey AK (2003). The influence of morphine on the absorption of paracetamol from various formulations in subjects in the supine position, as assessed by TDx measurement of salivary paracetamol concentrations. J Pharm Pharmacol.

[25] Holmér Pettersson P, Owall A, Jakobsson J (2004). Early bioavailability of paracetamol after oral or intravenous administration. Acta Anaesthesiol Scand.

[26] Moller PL, Sindet-Pedersen S, Petersen CT, Juhl GI, Dillenschneider A, Skoglund LA (2005). Onset of acetaminophen analgesia: comparison of oral and intravenous routes after third molar surgery. Br J Anaesth.

[27] Macario A, Royal MA (2011). A literature review of randomized clinical trials of intravenous acetaminophen (paracetamol) for acute postoperative pain. Pain Pract.

[28] Chiam E, Weinberg L, Bellomo R (2015). Paracetamol: a review with specific focus on the haemodynamic effects of intravenous administration. Heart Lung Vessel.

[29] Mahajan L, Mittal V, Gupta R, Chhabra H, Vidhan J, Kaur A (2017). Study to compare the effect of oral, rectal, and intravenous infusion of paracetamol for postoperative analgesia in women undergoing cesarean section under spinal anesthesia. Anesth Essays Res.

[30] Soyer T, Büyükkoçak Ü, Cesur Ö, Pekuz YÖ, Çakmak M (2008). Comparison of rectal and parenteral paracetamol administration in pain control after circumcision. KÜ Tıp Fak Derg.

[31] Anderson B, Kanagasundarum S, Woollard G (1996). Analgesic efficacy of paracetamol in children using tonsillectomy as a pain model. Anaesth Intensive Care.

[32] Zaki HA, Iftikhar H, Shallik N, Elmoheen A, Bashir K, Shaban EE, Azad AM (2022). An integrative comparative study between ultrasound-guided regional anesthesia versus parenteral opioids alone for analgesia in emergency department patients with hip fractures: a systematic review and meta-analysis. Heliyon.

[33] Zaki HA, Ibrahim T, Osman A, Elnabawy WA, Gebril A, Hamdi AH, Mohamed EH (2023). Comparing the safety and effectiveness of ketamine versus benzodiazepine/opioid combination for procedural sedation in emergency medicine: a comprehensive review and meta-analysis. Cureus.

[34] Boyle M, Nicholson L, O'Brien M, Flynn GM, Collins DW, Walsh WR, Bihari D (2010). Paracetamol induced skin blood flow and blood pressure changes in febrile intensive care patients: an observational study. Aust Crit Care.

